# Prevention and treatment of venous thromboembolism with low-molecular-weight heparins: Clinical implications of the recent European guidelines

**DOI:** 10.1186/1477-9560-6-13

**Published:** 2008-09-09

**Authors:** Paolo Prandoni

**Affiliations:** 1 Department of Cardiothoracic and Vascular Sciences, Thromboembolism Unit, University of Padua, Italy

## Abstract

Venous thromboembolism (VTE) is an important cause of avoidable morbidity and mortality. However, routine prophylaxis for at-risk patients is underused. Recent guidelines issued by an international consensus group, including the International Union of Angiology (IUA), recommend use of low-molecular-weight heparins (LMWHs) for the treatment of acute VTE and prevention of recurrence, and for prophylaxis in surgical and medical patients. This review highlights current inadequacies in the provision of thromboprophylaxis, and considers the clinical implications of the European guidelines on the prevention and treatment of VTE.

## Introduction

### The importance of preventing VTE

Venous thromboembolism (VTE), which includes deep vein thrombosis (DVT) and pulmonary embolism (PE), is an important cause of avoidable morbidity and mortality. Generally, massive PE occurs without warning. For patients dying in hospital as a result of PE, a diagnosis of PE has not even been considered in 70–80% of cases prior to death [1-4]. Although PE is a well-known complication of surgery, the risk is often underestimated in non-surgical patients, and approximately 75% of hospitalised patients suffering a fatal PE are actually medical patients [5,6]. Patients surviving an initial VTE event are at increased risk of recurrence [7], and are also at risk from considerable morbidity due to chronic venous insufficiency and pulmonary hypertension, which reduce quality of life and increase healthcare costs [8-10].

There is a strong association between asymptomatic DVT and the subsequent development of symptomatic VTE [11]. However, routine screening of patients for asymptomatic DVT is logistically problematic and is neither effective in preventing clinically significant VTE nor cost effective [12,13]. Consequently, prophylaxis is the most effective way of reducing morbidity and mortality among susceptible patients. Current clinical guidelines provide recommendations for thromboprophylaxis for many groups of hospitalised patients [12,13]. However, despite the widely accepted benefits, routine thromboprophylaxis for at-risk patients remains underused [14-18]. An international consensus group, including the International Union of Angiology (IUA), has recently issued detailed guidelines for the prevention and treatment of VTE [13]. The recommendations for prophylaxis have been based on a comprehensive review of published, peer-reviewed reports of randomised comparisons of different methods of thromboprophylaxis. They are fully consistent with those recently delivered by the American College of Chest Physicians [12].

This review aims to highlight the need for greater efforts in preventing VTE and considers the clinical implications of the recent European guidelines on the prevention and treatment of VTE.

### Which patients should be considered for routine thromboprophylaxis?

Risk factors for VTE are well known (Table 1). Patients with VTE generally have two or more risk factors, and the effects of multiple risk factors on VTE risk are additive. The type and duration of prophylaxis depends on whether the risk factors are transient (e.g., trauma, surgery, infection, the postpartum period) or persistent (e.g., advanced age, obesity, history of VTE, thrombophilia). Patients admitted to hospital are at particular risk of VTE, and the risk remains elevated after discharge [19,20]. This is particularly important in view of the current trend towards reducing the duration of inpatient stay, and suggests that patients will increasingly be discharged while still at risk. Furthermore, clinical events occurring after discharge from hospital can give the false impression of a declining risk of VTE related to hospitalisation.

**Table 1:** Risk factors for venous thromboembolism [13]

• Immobility	• Malignancy
• Trauma	• History of VTE
• Surgery	• Varicose veins
• Infection	• Dehydration
• The postpartum period	• Hormone therapy
• Advanced age	• Cancer therapies
• Obesity	• Thrombophilia
• Acute medical illness	

VTE, venous thromboembolism

The risks of VTE in surgical patients and the need for appropriate thromboprophylaxis have long been recognised. The risks are particularly high for orthopaedic surgery, where routine use of thromboprophylaxis is standard practice. Non-orthopaedic surgical patients are classified as being at high, medium or low risk of developing VTE on the basis of known risk factors (see additional file 1), and receive prophylaxis according to their level of risk [13]. However, patients hospitalised for an acute medical illness are also at increased risk for VTE. Hospitalisation for an acute medical illness has been shown to be independently associated with an approximately 8-fold increased relative risk for VTE [21]. The European guidelines highlight the fact that acute medical conditions such as stroke, congestive heart failure, respiratory disease, infections or myocardial infarction are associated with a high risk of VTE. The risk of VTE may be further increased by reduced mobility, cancer, or patient-related factors such as previous VTE, advancing age, obesity and coagulation disorders [13]. These risk factors are similar to those discussed in the recent guidelines on the prevention of VTE issued by the American College of Chest Physicians (ACCP) [12]. Both the European and ACCP guidelines recommend assessment of all hospitalised medical patients for risk of VTE so that appropriate thromboprophylaxis can be provided [12,13].

Patients with cancer are at particular risk from VTE, and it has been estimated that they have a 6-fold higher risk for VTE than non-cancer patients [21]. It is notable that PE is the second commonest cause of death in cancer patients, the first cause being the cancer itself [22]. Malignancy is associated with a hypercoagulable state, and several additional risk factors for VTE may co-exist in cancer patients [23]. Thromboprophylaxis is recommended for surgical cancer patients and for hospitalised cancer patients confined to bed with an acute illness [13]. Although cancer therapies have been associated with an increased risk of VTE [24,25], there is currently insufficient evidence to recommend prophylaxis routinely in ambulant, non-surgical cancer patients [13].

### How should VTE be prevented in at-risk patients?

Prevention is preferable to treatment of VTE because early symptoms and signs are unreliable predictors of clinically significant thromboembolic events, and fatal PE can occur without warning [12,13]. The approaches for prevention and treatment of VTE recommended in the recent European guidelines are based on published evidence in each clinical situation. The recommendations are graded (A-C) based on the level of supporting clinical evidence (Table 2) [13].

**Table 2:** Grades of recommendation in the IUA guidelines [13]

Grade	Clinical evidence
A	• Level 1 evidence from randomised controlled trials with consistent results, which are directly applicable to the target population.
B	• Level 1 evidence from a single high-quality randomised controlled trial, which is directly applicable to the target population.
	• Level 1 evidence from randomised controlled trials with less consistent results, limited power or other methodological problems, which are directly applicable to the target population.
	• Level 1 evidence from randomised controlled trials extrapolated from a different group of patients to the target population.
C	• Level 2 evidence from well-conducted observational studies with consistent results, which are directly applicable to the target population.

IUA, International Union of Angiology

Following a review of available evidence, the European consensus group concluded that there was no major difference between low-dose unfractionated heparin (LDUH) and low-molecular-weight heparins (LMWHs) when used for prophylaxis in general surgical patients [13]. LDUH or LMWH are recommended for general surgical patients identified as being at moderate or high risk, with prophylaxis commencing prior to the operation and continuing postoperatively (Grade A). An alternative approach for moderate-risk patients, particularly those in whom the risk of bleeding may be high, involves mechanical methods of prophylaxis (i.e., graduated elastic compression stockings and intermittent pneumatic compression) until the patient is ambulant (Grade A). Mechanical methods may also be used in conjunction with LDUH or LMWH in high-risk patients (Grade B). Use of fondaparinux in high-risk patients is a Grade B recommendation. In the absence of evidence from prospective clinical trials, these recommendations may be extrapolated to patients undergoing vascular or bariatric surgery (Grade C).

The European guidelines recommend specific prophylactic approaches for each type of orthopaedic surgery [13]. For example, for patients undergoing elective hip replacement, the recommended approach involves LMWH, fondaparinux or oral anticoagulant therapy, in addition to mechanical methods of prophylaxis (Grade A). Prophylaxis should be continued for 4–6 weeks with LMWH (Grade A) or fondaparinux (Grade C). For patients undergoing elective knee joint replacement, recommendations include LMWH or warfarin (Grade A), fondaparinux (Grade B) and mechanical methods (Grade B). Patients undergoing hip fracture surgery are at extremely high risk for DVT and fatal PE, and LMWH, fondaparinux, oral anticoagulant therapy or LDUH are recommended (Grade A). Throughout the guidelines, the IUA consensus group emphasises that mechanical methods should be considered whenever pharmacological prophylaxis is contraindicated.

### What treatment approaches are recommended for VTE?

Patients with VTE receive anticoagulants to treat the acute event and prevent fatal PE, and also to minimise the risks of developing post-thrombotic syndrome and recurrent VTE. For many years, unfractionated heparin (UFH) has been the standard treatment for acute VTE. However, clinical trial data show that LMWHs are more effective than UFH for the initial treatment of VTE and are associated with less major bleeding [26,27]. As a consequence, LMWHs are replacing UFH in the treatment of acute VTE. The recent European guidelines recommend that LMWH should be used in the initial treatment of VTE, followed by oral anticoagulant therapy for 3 months, or longer in the case of idiopathic VTE (Grade A) [13]. These recommendations are fully consistent with those recently delivered by the American College of Chest Physicians [28].

On the basis of data from two clinical trials [29,30], fondaparinux may be used as an alternative to LMWH.

In patients with cancer, the IUA guidelines recommend either LMWH or UFH for initial treatment of VTE (Grade A), but note that outpatient therapy with LMWH is preferred, particularly in patients with a reduced life expectancy in whom quality of life becomes a priority [13]. Recent studies have shown home treatment with LMWH to be feasible in the majority of patients with cancer and acute VTE [31,32]. For prevention of recurrent VTE in patients with cancer, prophylaxis with the LMWH dalteparin is recommended for 6 months using the dosing regimen that was shown to be effective in a major clinical trial (Grade B) [33]. This regimen involves intensive initial anticoagulation for 1 month (200 IU/kg once daily) followed by less intensive anticoagulation for 5 months (150 IU/kg once daily) to reduce the risk of bleeding. In view of the ongoing risk of VTE in patients with cancer, continued treatment should be considered for as long as the cancer is active or while patients are receiving anticancer therapy (Grade C) [13].

### Is there any difference between individual LMWHs?

LMWHs are manufactured by a variety of different methods, and each LMWH has a unique chemical structure that confers specific pharmacokinetic and pharmacodynamic properties. Regulatory bodies in Europe and North America regard LMWHs as distinct chemical entities, and advise that they should not be used interchangeably [13,34]. Very little is known about the relative efficacy and safety of different LMWHs in the treatment and prevention of VTE. The few direct comparative trials of LMWHs that have been conducted in the treatment of acute VTE [35] and thromboprophylaxis [36-39] have not revealed any notable differences in clinical efficacy or safety between individual LMWHs.

In the absence of sufficient comparative data, each LMWH should be dosed according to the labelling and recommendations issued by the manufacturer. Thus, the clinical results from one LMWH should not be extrapolated to another. Adopting an evidence-based approach to patient management is essential in the use of LMWHs, and only doses of drugs that have been adequately evaluated in well-designed clinical trials should be considered for use in a particular indication.

## Conclusion: improving compliance with clinical guidelines

Despite the existence of comprehensive consensus guidelines for the prevention and treatment of VTE [7,12,13], thromboprophylaxis remains underused [14-18]. Reasons for underuse are complex and include underestimation of the risks of VTE, underestimation of the impact of non-fatal outcomes of VTE, lack of awareness of relevant guidelines, absence of local thromboprophylaxis strategies, and concerns about the risk of bleeding. Concerns about the risk of bleeding are generally unfounded, as the risk of bleeding complications with LMWHs is low [12,13]. Nonetheless, this is often cited as a reason for underuse of thromboprophylaxis, particularly in surgical patients [12,16,39].

Improving compliance with thromboprophylaxis guidelines is a complex task that needs to include improved thrombotic risk-assessment methods, familiarisation of clinicians with current best practice, and facilitation of appropriate prescribing of prophylaxis [39]. Several simple and clinically relevant risk assessment models are now available to assist with VTE risk stratification of hospitalised patients [6,40,41]. Recent risk assessment models in medical and surgical patients have also included suggested thromboprophylaxis strategies [6,41]. Evidence suggests that the prevention of VTE can be improved by use of computerised reminders for clinicians to consider prophylaxis in at-risk patients [42,43]. Although these initiatives go some way towards improving the prescribing of thromboprophylaxis, additional efforts are still needed to increase the routine use of thromboprophylaxis in patients at risk of VTE to further reduce the morbidity and mortality of this preventable condition.

## Competing interests

The author declares that they have no competing interests.

## Supplementary Material

Additional file 1 Risk categories in non-orthopaedic surgical patients [13]. Reproduced with permission from the Cardiovascular Disease Educational and Research Trust. Risk of postoperative VTE in non-orthopaedic surgical patients, according to patients' characteristics and type of surgical operation. Click here for file
