# Supplementary material for: Prevention and treatment of venous thromboembolism with low-molecular-weight heparins: Clinical implications of the recent European guidelines
Source: Thromb J. 2008 Sep 9;6:13. doi: 10.1186/1477-9560-6-13 (PMC2546366; doi:10.1186/1477-9560-6-13)
Supplement: Additional file 1 — Risk categories in non-orthopaedic surgical patients [13]. Reproduced with permission from the Cardiovascular Disease Educational and Research Trust. Risk of postoperative VTE in non-orthopaedic surgical patients, according to patients' characteristics and type of surgical operation. [file 1477-9560-6-13-S1.doc]

**Additional file**

Risk categories in non-orthopaedic surgical patients [13] Reproduced with permission from the Cardiovascular Disease Educational and Research Trust

|  | Level of risk | | |
| --- | --- | --- | --- |
|  | High | Moderate | Low |
| General surgery | Major general surgery, age >60 | Major general surgery, age 40–60 without other risk factors** | Major general surgery, age <40 without other risk factors** |
|  | Major general surgery, age 40–60 with cancer or prior VTE | Minor surgery, age >60 | Minor surgery, age 40–60 without other risk factors** |
|  | Thrombophilia | Minor surgery, age 40–60 with prior VTE or receiving oestrogen therapy |  |
| Gynaecology | Major gynaecological surgery, age >60 | Major gynaecological surgery, age 40–60 | Minor gynaecological surgery, age <40 without other risk factors** |
|  | Major gynaecological surgery, age 40–60 with cancer or prior VTE | Major gynaecological surgery, age <40 and receiving oestrogen therapy | Minor gynaecological surgery, age 40–60 without other risk factors** |
|  | Thrombophilia | Minor surgery, age >60 |  |
| Obstetrics* | Prior VTE | Age >35, obese and undergoing Caesarian section | Age <35 without any risk factors |
|  | Thrombophilia |  |  |
| Frequencies of VTE complications in the absence of prophylaxis (%): |  |  |  |
| Calf DVT | 40–80 | 10–40 | <10 |
| Proximal DVT | 10–30 | 1–10 | <1 |
| Fatal PE | >1 | 0.1–1 | <0.1 |
| * The risk of DVT in obstetric patients with preeclampsia and the other factors is unknown but prophylaxis should be considered;  ** The risk is increased by infectious disease, presence of varicose veins and general immobility;  Minor surgery: operations other than abdominal lasting <45 min;  Major surgery: any intra-abdominal operation and all other operations lasting >45 min;  DVT, deep vein thrombosis;  PE, pulmonary embolism. | | | |
